# AI-Based Pulmonary Embolism Detection: The Added Value of a False-Positive Reduction Module over a Region Proposal Network

**DOI:** 10.3390/diagnostics16040524

**Published:** 2026-02-09

**Authors:** Jeong Sub Lee, Euijin Hwang, Changgyun Jin, Kyong Joon Lee, Ye Ra Choi, Sang Il Choi

**Affiliations:** 1 College of Medicine, Seoul National University, Seoul 03080, Republic of Korea; dlwjd023677@gmail.com; 2 Department of Radiology, Seoul National University Hospital, Seoul 03080, Republic of Korea; ken92002@snu.ac.kr; 3 Monitor Corporation, Seoul 06628, Republic of Korea; cg.jin@monitorcorp.ai (C.J.); kjoon31@gmail.com (K.J.L.); 4 Department of Radiology, Seoul National University Bundang Hospital, Seongnam-si 13620, Republic of Korea; 5 Department of Radiology, Seoul Metropolitan Government-Seoul National University Boramae Medical Center, Seoul 07061, Republic of Korea

**Keywords:** pulmonary embolism, artificial intelligence, deep learning, computed tomography angiography, false positive reduction, Mask Region-based Convolutional Neural Network

## Abstract

**Background**: High false-positive rates remain a significant challenge in the automated detection of pulmonary embolism (PE) using Computed Tomography Pulmonary Angiography (CTPA). This study evaluated the additional value of a False-Positive Reduction (FPR) module integrated into a Region Proposal Network (RPN). **Methods**: A retrospective analysis of 303 CTPA scans (163 PE-positive and 140 PE-negative) was conducted from a single tertiary institution. Both models were additionally validated on an independent external cohort of 100 CTPA scans (50 PE-positive and 50 PE-negative) from the RSNA PE Challenge dataset. The diagnostic performance of the one-stage RPN-only model was compared with that of a two-stage Modified Mask R-CNN (Region-based Convolutional Neural Network) incorporating the FPR module. **Results**: The Modified Mask R-CNN exhibited significant improvement in terms of specificity. The false-positive rate per scan decreased by 31% in comparison to the RPN-only model. Although there was a slight reduction in patient-level sensitivity, the Positive Predictive Value significantly increased by 10.5%. Additionally, patient-level specificity for emboli with a volume ≥ 1000 mm^3^ increased, reflecting a 7.4% relative improvement in detecting clinically significant emboli. **Conclusions**: The Modified Mask R-CNN significantly reduced false positives while maintaining high sensitivity over a region proposal network.

## 1. Introduction

Pulmonary Embolism (PE) is the third most frequent acute cardiovascular event, with an estimated annual incidence ranging from 39 to 115 per 100,000 individuals [1,2]. The clinical spectrum of PE ranges from asymptomatic subsegmental emboli to massive life-threatening emboli with hemodynamic instability. Prompt recognition and immediate initiation of appropriate treatment remain critical, as delayed diagnosis significantly increases pulmonary embolism-related morbidity and mortality. Untreated PE has a mortality rate approaching 30%, whereas timely anticoagulation reduces this rate to approximately 2–8% [1,2]. The diagnostic challenge is further compounded by the nonspecific clinical presentation of PE, which frequently mimics other cardiopulmonary conditions, necessitating the use of advanced imaging modalities for a definitive diagnosis.

Computed Tomography Pulmonary Angiography (CTPA) has been established as the gold standard for diagnosing PE owing to its rapid acquisition time, high spatial resolution, and broad accessibility [3,4]. Since its introduction in the late 1990s, multi-detector CTPA has largely replaced ventilation-perfusion scintigraphy and invasive pulmonary angiography in diagnostic algorithms for suspected PE. However, the diagnostic accuracy of CTPA can be compromised by various factors, including reader variability, suboptimal contrast enhancement, and motion artifacts. Studies have demonstrated inter-observer agreement, with kappa values ranging from 0.81 to 0.89 for PE detection [5], indicating substantial but imperfect concordance, even among experienced radiologists. Consequently, the reported positivity rates of CTPA remain variable, ranging between 10% and 30% [6,7], with considerable institutional variation depending on patient selection criteria and clinical practice patterns.

The incorporation of Artificial Intelligence (AI), particularly Deep Learning (DL), into radiology has markedly enhanced the diagnostic efficacy of pulmonary embolism detection. Initial computer-aided detection (CAD) systems exhibited limited clinical utility because of elevated false-positive rates and computational limitations. In contrast, recent deep learning models, notably Convolutional Neural Networks (CNNs), have demonstrated high sensitivity and accuracy in the automated detection of PE from CTPA images [8,9,10,11,12]. A recent meta-analysis of 24 studies involving 22,984 patients reported pooled estimates of 89.4% sensitivity and 87.1% specificity for DL-based PE detection, highlighting significant architectural variations among model types [8,13]. While U-Net-based architectures achieved sensitivity of approximately 88.8% through pixel-level segmentation capabilities [10,13], CNN-based approaches demonstrated sensitivity of 88.7% and specificity of 89.1% through hierarchical feature extraction and robust classification [8,11,13]. These advancements have enabled the detection of small subsegmental emboli that may be overlooked by human readers, particularly in high-volume emergency department settings, where time constraints and reader fatigue can impair diagnostic performance [8,14]. FDA-cleared AI algorithms have demonstrated substantial potential for clinical applications [15].

However, a critical limitation persists: excessively high false-positive rates that compromise clinical utility. The meta-analysis revealed a pooled positive predictive value (PPV) of only 83.2% [13], meaning that approximately one in six positive detections represents a false alarm, imposing substantial interpretive burden on radiologists. False-positive detections, primarily caused by vascular mimics such as lymph nodes, venous flow artifacts, beam-hardening effects, and partial-volume averaging, can result in unnecessary follow-up imaging, patient anxiety, and inappropriate initiation of anticoagulation therapy with associated bleeding risks [16,17]. Studies have reported false-positive rates ranging from 8% to over 35% per scan [11,18], with marked heterogeneity across studies (I^2^ ≈ 97%) [13], suggesting that existing approaches lack robust mechanisms to consistently suppress false positives in diverse clinical settings.

Previous research in medical imaging has addressed false positives through geometric feature engineering and attention mechanisms, such as multi-view convolutional networks for pulmonary nodule detection [19] and dual-branch architectures for lesion segmentation [20]. Recent studies have also explored probability-based Mask R-CNN variants [18] and 3D CNN architectures for PE detection [21], achieving promising sensitivity metrics. However, a critical gap remains in the literature: existing approaches typically lack a dedicated two-stage architecture that combines high-sensitivity initial detection with a specialized false-positive reduction module. Specifically, no prior study has integrated HU-based attenuation analysis with 3D morphological feature learning to systematically address venous flow artifacts and anatomical mimics, which are the primary sources of false-positive detections that compromise the clinical utility of automated PE detection systems [8,16]. While sensitivity improvements have been the primary focus of recent developments, persistently high false-positive rates [11,18] remain a fundamental barrier to clinical adoption, as they impose an additional interpretive burden on radiologists rather than reducing it [22].

To address this challenge, we developed a two-stage Modified Mask R-CNN framework [23] that achieves high sensitivity while significantly reducing false-positive rates—a critical requirement for clinical workflow efficiency [11,18]. In Stage 1, we employed DuckNet [24], a U-Net variant that integrates convolutional neural network techniques into its encoder–decoder architecture. This design leverages both the pixel-level segmentation capabilities of U-Net for precise lesion boundary delineation and CNN’s hierarchical feature extraction for robust pattern recognition, enabling the comprehensive detection of potential embolic lesions with high recall. The DuckNet-based Region Proposal Network (RPN) [25] generates candidate regions that capture even small subsegmental emboli that might be missed by conventional detection approaches. In Stage 2, a dedicated False-Positive Reduction (FPR) module filters these candidates using Hounsfield Unit (HU)-based attenuation analysis combined with 3D morphological features [26]. This HU-based classification effectively discriminates true emboli from common vascular mimics—such as lymph nodes, contrast artifacts, and venous flow patterns—that exhibit characteristic density differences on CTPA. The two-stage design allows independent optimization: Stage 1 maximizes sensitivity through comprehensive candidate detection, and Stage 2 enhances precision by eliminating false positives based on the quantitative imaging features. This balanced approach addresses the fundamental limitation of existing systems that prioritize either sensitivity or specificity at the expense of the other factors [8,27].

## 2. Materials and Methods

### 2.1. Study Population and Data Acquisition

This retrospective study was approved by the Institutional Review Board (IRB) of Seoul National University Bundang Hospital (No. B-2406-908-104), and the requirement for informed consent was waived owing to the retrospective nature of the study. We reviewed the Computed Tomography Pulmonary Angiography (CTPA) examinations performed between January 2022 and December 2024 at a single tertiary institution (Seoul National University Bundang Hospital). A total of 354 patients who underwent CTPA during this period were initially selected based on the inclusion criteria: individuals who underwent contrast-enhanced CTPA and were suspected of or diagnosed with pulmonary embolism. The inclusion criteria required patients to be at least 18 years of age with technically adequate CTPA studies (main pulmonary artery attenuation ≥ 200 Hounsfield Units). The exclusion criteria were unrelated clinical indications, poor image quality precluding diagnostic interpretation, and extensive parenchymal abnormalities that could interfere with the automated detection algorithms.

### 2.2. Training and Internal Validation Sets

**Figure 1:**
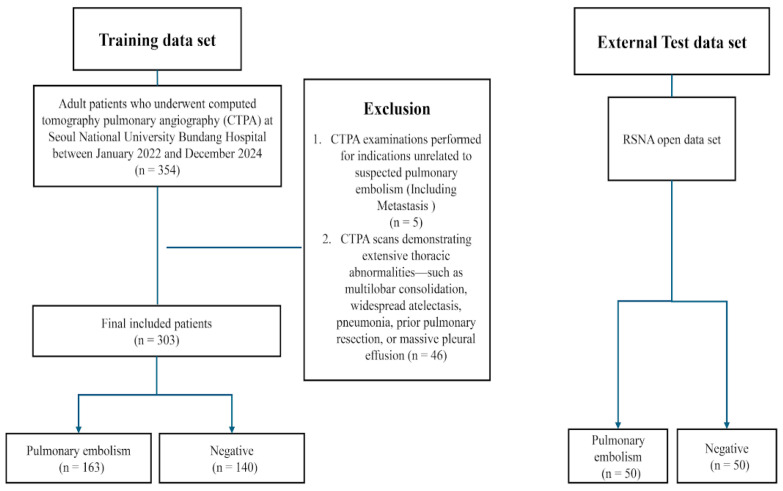
Flowchart of the study population inclusion and exclusion process.

The training dataset was derived from a real-world clinical cohort consisting of 354 CTPA examinations. Following the application of exclusion criteria (n = 51; including five unrelated indications, five of poor quality, and 41 with extensive abnormalities), the final training set comprised 303 CTPA scans (163 PE-positive and 140 PE-negative). The detailed patient selection process and exclusion criteria are depicted in Figure 1. Ground-truth annotations were performed using the Redbrick AI platform (Redbrick AI, Wilmington, DE, USA), a cloud-based system optimized for 3D volumetric imaging. Two board-certified thoracic radiologists, each possessing over 10 years of experience, independently reviewed each case. Discrepancies were resolved through consensus review moderated by a senior thoracic radiologist.

The internal dataset was randomly divided into training (n = 243, 80%) and validation (n = 60, 20%) subsets using stratified sampling at the patient level to prevent data leakage and ensure a balanced representation of PE-positive and PE-negative cases in both subsets. The validation set was used for hyperparameter tuning and model selection, and the final model performance was assessed using an independent external test set.

### 2.3. External Test Set

To evaluate the generalizability of the model, an independent external test cohort comprising 100 CTPA scans (50 PE-positive and 50 PE-negative) was retrospectively curated from the publicly available RSNA PE Challenge dataset. Consistent with the internal dataset, cases with inadequate contrast (Hounsfield Unit (HU) ≤ 200) or severe artifacts were excluded. All external cases were reannotated by two board-certified radiologists (with 9 and 12 years of experience) to ensure consistent labeling standards. Although the slice thickness was standardized to 3 mm, this dataset encompassed a wide spectrum of CT scanner vendors and reconstruction kernels, reflecting real-world heterogeneity.

### 2.4. CT Acquisition Protocol

All CTPA examinations within the internal cohort were conducted using 128-slice multi-detector CT scanners from two manufacturers: Siemens SOMATOM Definition Flash (Siemens Healthcare, Erlangen, Germany) and Philips Brilliance iCT (Philips Healthcare, Best, The Netherlands). To ensure uniformity in image quality and facilitate standardized analysis, only examinations with a slice thickness of 3.0 mm were included in this study. The scanning parameters comprised a tube voltage of 80–140 kV, adjusted according to the body mass index, and automatic tube current modulation (CARE Dose4D for Siemens; DoseRight for Philips), with reference values of 80–150 mAs.

Automated bolus tracking was employed, with the region of interest positioned in the main pulmonary artery, triggered at 100 Hounsfield Units (HU). Image quality was assessed by measuring pulmonary artery enhancement, and examinations with mean attenuation below 200 HU were excluded to ensure adequate contrast.

For external validation, the publicly available RSNA-STR Pulmonary Embolism CT (RSPECT) Dataset was utilized, compiled for the 2020 RSNA Pulmonary Embolism Detection Challenge. This dataset comprises over 12,000 CTPA examinations from five international research centers, with expert annotations provided by more than 80 thoracic radiologists. The dataset exhibited substantial heterogeneity, with scans acquired using various CT systems from General Electric, Siemens, and Philips, and tube voltages ranging from 80 to 140 kV. Consistent with our internal cohort, only examinations with a slice thickness of 3.0 mm were selected from this dataset to maintain uniformity in the spatial resolution across both the training and validation cohorts.

### 2.5. Algorithm Architecture

**Figure 2:**
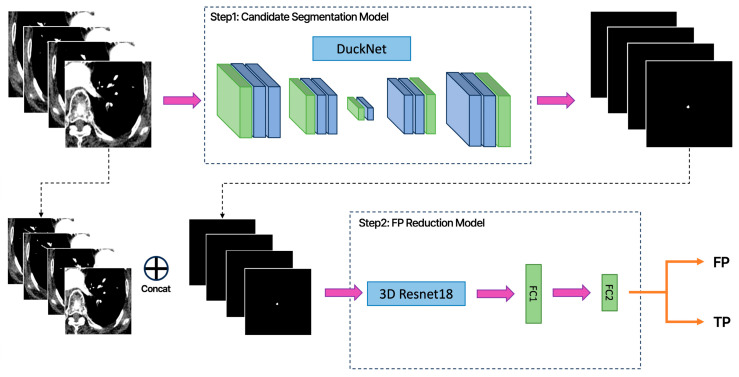
Schematic overview of the proposed two-stage deep learning framework. The pipeline consists of two sequential stages for automated pulmonary embolism detection. Stage 1 (Candidate Generation) utilizes a DuckNet architecture to process 2D slices from the resampled CT volume, generating initial binary masks with high sensitivity. Stage 2 (False-Positive Reduction) extracts 3D volumetric patches corresponding to the candidate masks.

We propose a cascaded two-stage deep learning framework (Figure 2) for the automated detection of PE. A one-stage RPN-only model served as the baseline, while a two-stage Modified Mask R-CNN was utilized as the proposed method. Both architectures were implemented within the LuCAS-EMB framework (Lung Cancer Screening and Embolism Detection System, MLB-01 platform; Monitor Corporation, Seoul, Republic of Korea). For model optimization, the networks were trained using the AdamW optimizer with a learning rate ranging from 1 × 10^−6^ to 5 × 10^−3^, regulated by a Cosine Annealing Warm-up scheduler. The training process was conducted with a total batch size of 14, distributed across 10 NVIDIA A40 GPUs (NVIDIA Corporation, Santa Clara, CA, USA) to ensure stable convergence and computational efficiency.

**Figure 3:**
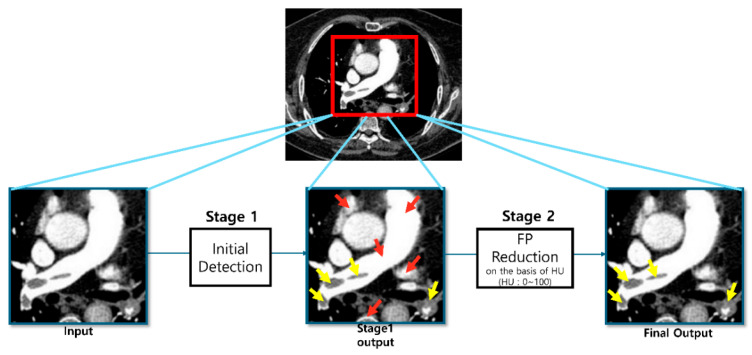
Illustration of the two-stage Modified Mask R-CNN framework. Stage 1 generates initial candidates (red and yellow arrows) with high sensitivity. Stage 2 employs a False-Positive Reduction (FPR) module to analyze 3D morphological features, effectively eliminating false positives (red arrows) while retaining true emboli (yellow arrows).

- Stage 1 (RPN-Only Model): The baseline architecture (LuCAS-EMB, MLB-01, v1.00.00, Monitor Corporation, Republic of Korea) employed a DuckNet [24] architecture, which is a U-Net-based model [28], to process volumetric CTPA data and generate binary masks for all suspicious regions. The primary objective was to achieve a high recall to minimize missed true emboli, despite the inherent generation of false positive (FP) candidates.
- Stage 2 (False-Positive Reduction): To improve precision, a dedicated FP reduction model (LuCAS-EMB, MLB-01, Research version, Monitor Corporation, Republic of Korea) was introduced (Figure 3). For each candidate mask, a corresponding 3D volumetric patch was extracted and fed into a 3D ResNet18 [26]. This architecture leverages the inter-slice spatial context to learn 3D morphological features distinguishing true emboli from mimics (e.g., hilar lymph nodes and partial volume artifacts). During the training phase, a balanced data distribution was maintained with a 1:1 ratio between True Positive and False Positive samples to prevent model bias. The network includes two fully connected layers to classify candidates into True Positives (TP) and False Positives (FP). To achieve optimal classification performance, the decision threshold for distinguishing TPs from FPs was empirically determined and fine-tuned based on the results of the validation set.

The FPR module employs a volume-based filtering strategy with a threshold of 1000 mm^3^. Notably, this threshold primarily serves a technical and statistical role for performance optimization and standardized comparison with prior deep learning studies, rather than representing a strict clinical cutoff for treatment decisions. This threshold has been utilized in previous deep learning-based PE detection studies to achieve an optimal balance between sensitivity (0.88) and specificity (0.88) for patient-level PE detection [11,22], providing a standardized benchmark for algorithmic performance comparison across studies.

### 2.6. Evaluation Metrics

Model performance was assessed using comprehensive metrics at both the lesion and patient levels to provide a clinically relevant evaluation. For lesion-level evaluation, a predicted embolus was considered a true positive if its centroid fell within the ground-truth mask. Lesion-level metrics included sensitivity and false-positive rates per scan (FPRs/scan), which were calculated as the total number of false-positive detections divided by the total number of scans.

For patient-level evaluation, a patient was classified as PE-positive if at least one embolus was detected anywhere in the pulmonary arterial tree. Patient-level sensitivity, specificity, and positive predictive value (PPV) were computed.

### 2.7. Statistical Analysis

The diagnostic performance was evaluated using sensitivity, specificity, positive predictive value (PPV), and false-positive rates per scan (FP/scan) at both the lesion and patient levels. For proportion-based metrics, 95% confidence intervals were calculated using the Wilson score method. To compare the performance of the RPN-only model and the Modified Mask R-CNN, McNemar’s test was used for paired binary classification outcomes (sensitivity and specificity), and the Wilcoxon signed-rank test was used to compare false-positive rates per scan. A two-sided *p*-value of < 0.05 was considered statistically significant. All analyses were conducted using R software (version 4.3.2; R Foundation for Statistical Computing, Vienna, Austria).

## 3. Results

This section presents a comprehensive evaluation of the proposed Modified Mask R-CNN compared to the baseline RPN-only model. We report the baseline characteristics of the study population, followed by a detailed analysis of the diagnostic performance of both the internal validation dataset and independent external test cohort.

### 3.1. Study Population and Baseline Characteristics

A total of 303 patients were included in the internal training and validation cohorts. The flow of the patient selection and exclusion criteria is shown in Figure 1. The detailed demographic and clinical characteristics are summarized in Table 1. The mean age was 63.2 ± 16.8 years, with 161 female (53.1%) and 142 male (46.9%) patients. No significant differences were observed between PE-positive (n = 163) and PE-negative (n = 140) groups in age (*p* = 0.78), sex distribution (*p* = 0.158), hypertension (*p* = 0.71), diabetes mellitus (*p* = 0.48), or active malignancy (*p* = 0.18).

D-dimer levels were significantly elevated in patients with PE (median 1.20 mg/L, IQR: 0.55–3.40) compared to those without PE (median 0.42 mg/L, IQR: 0.26–0.82, *p* < 0.001). The majority of examinations were performed using Philips CT scanners (81.2%), with the remainder performed using Siemens Healthineers equipment (18.8%). Scanner distribution did not differ significantly between PE-positive and PE-negative groups (*p* = 0.069).

### 3.2. Performance on the Internal Validation Dataset

**Figure 4:**
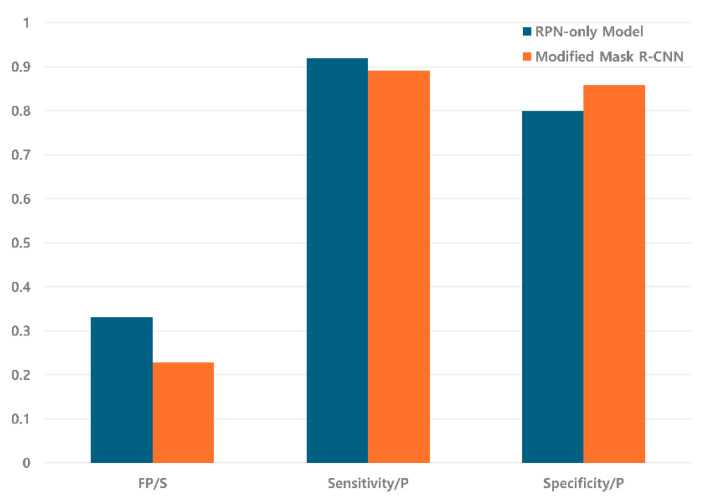
Comparative performance on the internal validation dataset. The Modified Mask R-CNN (orange) achieved a substantial reduction in false-positive rates per scan compared to the RPN-only model (blue), while maintaining high patient-level sensitivity.

The comparative diagnostic performances of the RPN-only model and the Modified Mask R-CNN on the internal validation dataset are detailed in Table 2. At the patient level, the Modified Mask R-CNN achieved a sensitivity of 0.892, reflecting a slight decrease from the RPN-only model’s sensitivity of 0.92 (difference: −0.028). Despite this minor reduction in sensitivity, the modified model exhibited significant improvements in precision metrics. The Modified Mask R-CNN attained a Positive Predictive Value (PPV) of 0.718, compared to 0.65 for the RPN-only model, indicating a 10.5% relative enhancement in diagnostic precision. Additionally, the false-positive rate per scan decreased from 0.331 to 0.228, representing a 31% reduction in false-positive detections (Figure 4). This reduction in false positives was achieved while maintaining clinically acceptable sensitivity (0.892), demonstrating the efficacy of the FPR module in filtering false detections without substantially compromising true PE detection.

At the lesion level, the Modified Mask R-CNN achieved a sensitivity of 0.87 compared to 0.884 for the RPN-only model, with the lesion-level PPV improving from 0.573 to 0.658. This demonstrates the effective detection and characterization of individual embolic lesions. Notably, for emboli with a volume ≥1000 mm^3^, patient-level specificity improved from 0.8 in the RPN-only model to 0.859 in the Modified Mask R-CNN, representing a 7.4% relative enhancement. This improvement in specificity for larger, clinically significant emboli highlights the ability of the FPR module to effectively discriminate true emboli from vascular mimics and anatomical structures that commonly generate false-positive results. A volume threshold of 1000 mm^3^ was selected based on the established literature demonstrating optimal diagnostic performance at this cut-off for identifying hemodynamically significant emboli in deep learning-based detection systems [9,11].

**Figure 5:**
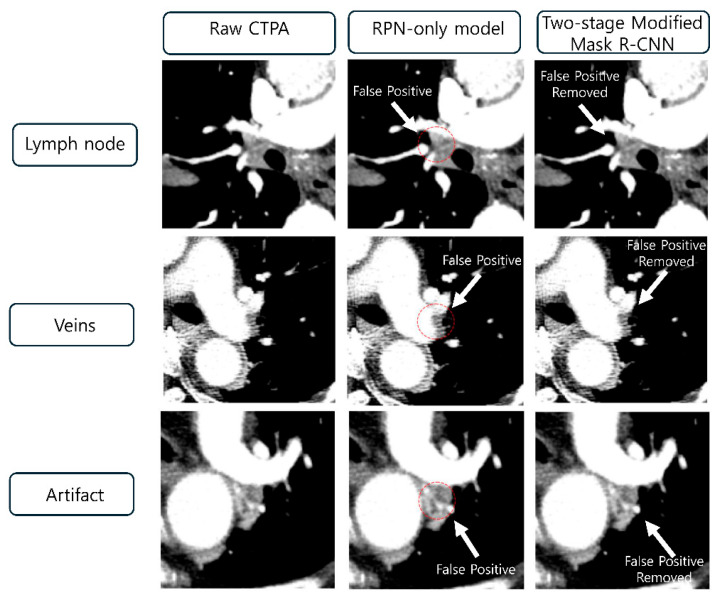
Representative examples of false-positive reduction by the Modified Mask R-CNN. Three common sources of false positives in automated pulmonary embolism detection are shown: hilar lymph nodes mimicking filling defects (**top row**), contrast-enhanced pulmonary veins (**middle row**), and beam-hardening artifacts near the superior vena cava (**bottom row**). Left column: Original axial CTPA images. Middle column: RPN-only model output with false-positive detections indicated by the red circles. Right column: Modified Mask R-CNN output demonstrating the successful elimination of false positives using the dedicated FPR module. The white arrows indicate the anatomical structures that were incorrectly identified by the RPN-only model but correctly filtered by the two-stage framework.

To illustrate the clinical utility of the FPR module, representative examples of false-positive reductions are shown in Figure 5. The figure demonstrates three common sources of false positives in automated PE detection: lymph nodes, veins, and imaging artifacts. In each case, the RPN-only model incorrectly identified these structures as potential emboli, as indicated by red circles. However, the two-stage Modified Mask R-CNN successfully eliminated these false detections through the FPR module, as evidenced by the “False Positive Removed” annotations. These examples highlight the model’s improved specificity in distinguishing true PE from anatomical mimics and artifacts, which is crucial for reducing unnecessary clinical workload and improving diagnostic accuracy. These results demonstrate that the FPR module successfully enhanced diagnostic precision while maintaining clinically acceptable sensitivity. The Modified Mask R-CNN achieved a 31% reduction in false positives, 10.5% improvement in PPV, and 7.4% enhancement in specificity for clinically significant emboli (volume ≥ 1000 mm^3^). The meaningful improvement achieved from an already reasonable baseline (RPN-only model with PPV = 0.65 and specificity = 0.8) demonstrates the effectiveness of the dedicated FPR stage, even when the initial detection model performs well.

### 3.3. Performance on the External Test Dataset (RSNA Cohort)

To assess the generalizability of the proposed framework, external validation was performed on an independent RSNA cohort (n = 100), which differed substantially from the internal cohort in several important aspects. First, while the internal cohort was acquired using only two CT scanner models (Siemens SOMATOM Definition Flash and Philips Brilliance iCT), the RSNA dataset encompassed CT systems from multiple vendors, including General Electric, Siemens, and Philips, reflecting greater real-world heterogeneity in acquisition parameters and image reconstruction kernels [2,4]. Second, the internal cohort represented consecutive patients from a single tertiary institution with standardized imaging protocols, whereas the RSNA dataset comprised cases from five international research centers with varying patient selection criteria and clinical practice patterns. Third, although both cohorts underwent expert radiologist annotation, the RSNA dataset was originally annotated by over 80 thoracic radiologists with inherently greater inter-annotator variability and was re-annotated by two board-certified radiologists for this study to ensure labeling consistency. Despite these substantial differences, the Modified Mask R-CNN demonstrated consistent performance improvements over the RPN-only model. The results are presented in Table 3.

On this heterogeneous dataset, the Modified Mask R-CNN exhibited substantial improvement in reliability. The mean number of false positives per scan was significantly reduced from 1.1 (RPN-only) to 0.34 (Modified Mask R-CNN), representing a 69% reduction in false-positive detections (from 1.1 to 0.34). Although a trade-off was observed with a decrease in patient-level sensitivity from 0.96 to 0.88, this was compensated by notable improvements in patient-level specificity for emboli ≥ 1000 mm^3^ (from 0.59 to 0.84) and PPV (from 0.52 to 0.76). These findings suggest that the Modified Mask R-CNN is far more effective in excluding non-embolic cases and provides reliable positive findings in a multicenter setting.

### 3.4. Qualitative Analysis

The two-stage detection pipeline effectively demonstrates the capability of the Modified Mask R-CNN framework to reduce false positives. As detailed in the Methods section, the initial detection module identified both true emboli and false-positive candidates. Subsequently, the dedicated false-positive reduction (FPR) module successfully eliminated erroneous detections, resulting in a final output that predominantly contains true embolic lesions.

**Figure 6:**
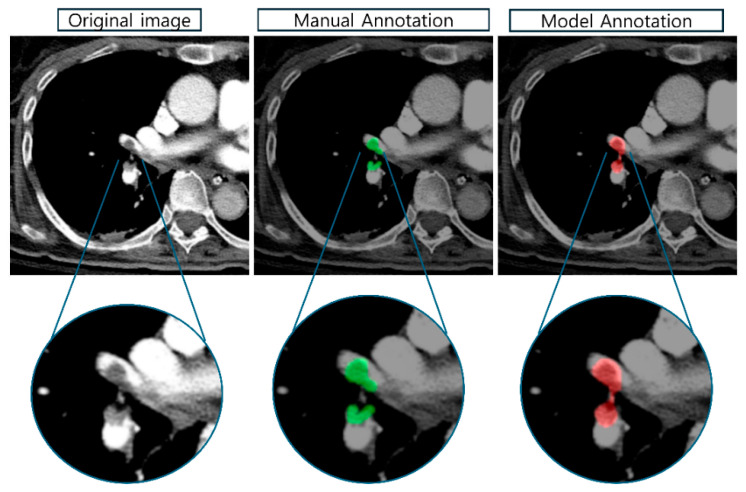
Segmentation accuracy comparison. Green contours represent expert radiologist annotations (Ground Truth), and red contours represent the model’s predictions. The Modified Mask R-CNN demonstrates high concordance with manual annotations, accurately delineating embolus boundaries even in complex anatomical locations like the main pulmonary artery bifurcation.

The model’s segmentation accuracy is further demonstrated in Figure 6, which compares manual annotations (green) with the model’s predictions (red). The Modified Mask R-CNN showed high concordance with expert radiologist annotations, accurately delineating embolus boundaries across various anatomical locations. The example shown depicts a saddle embolus in the main pulmonary artery bifurcation, where the model successfully captured the complex morphology including irregular borders and partial occlusions. Magnified views demonstrate pixel-level agreement between manual and automated segmentations.

To determine the optimal volume threshold for clinical application, we evaluated the Modified Mask R-CNN performance across different volume cut-offs ranging from 300 to 2000 mm^3^ (Table 4). The analysis demonstrated that sensitivity remained relatively stable (0.86–0.93) across all thresholds, while specificity and PPV improved with larger volume thresholds. The 1000 mm^3^ threshold provided a balanced trade-off between sensitivity (0.88), specificity (0.88), and false-positive rate (0.168 per scan), supporting its selection for our primary analysis.

## 4. Discussion

The principal finding of this study is that incorporating a False-Positive Reduction (FPR) module into a Modified Mask R-CNN architecture significantly enhances the specificity of Pulmonary Embolism detection on CTPA while maintaining high sensitivity. Compared to the baseline RPN-only approach, the Modified Mask R-CNN achieved a substantial reduction in false-positive rates without a statistically significant decline in sensitivity. This highlights the added value of a dedicated FPR module in effectively distinguishing true embolic lesions from radiological mimics, thereby improving diagnostic precision in clinical settings.

### 4.1. Methodological Advances and Distinction from Prior Work

Previous studies have successfully adapted Mask R-CNN architectures [24] to various medical imaging tasks, including pulmonary nodule detection [19,20,26] and polyp segmentation [24]. Specifically for pulmonary embolism detection, probability-based Mask R-CNN variants [19] have demonstrated improved performance in reducing false-positive rates. Some studies have implemented two-stage approaches to refine detection performance [19,20].

However, to the best of our knowledge, no study has specifically integrated a dedicated FPR module with 3D morphological feature learning. Unlike predominantly 2D approaches processing slices independently, our 3D ResNet18 captures volumetric characteristics—inter-slice continuity, vessel geometry, clot morphology [29,30]. This distinction is particularly relevant given the clinical importance of reliably minimizing diagnostic ambiguity, especially for less experienced readers [31]. Existing PE detection models remain vulnerable to persistently high false-positive rates, particularly those related to complex vascular anatomy and flow artifacts, which hinder their utility as reliable clinical decision-support tools [8,17]. Therefore, our study addresses the ongoing and substantial need for methods that focus on meaningfully reducing false-positive outputs, thereby facilitating clinical implementation and enhancing diagnostic performance.

The performance of our model falls within this established benchmark range while offering the added advantage of a modular FPR component that can be fine-tuned for specific clinical environments and scanner protocols. The consistency of our model’s performance across both internal and external cohorts suggests robust generalizability, which is a critical attribute for clinical deployment in diverse healthcare settings [32].

### 4.2. Clinical Implications and Workflow Integration

A significant finding of this study is that the Modified Mask R-CNN, initially developed to address false positives associated with vascular structures, also demonstrated considerable efficacy in reducing false positives from non-vascular structures. From a clinical perspective, this reduction in false-positive detections offers distinct advantages for pulmonary embolism diagnosis, particularly in settings where rapid and accurate decision-making is crucial. The reduction in false-positive rates may potentially support less experienced readers by minimizing interpretive ambiguity, although prospective validation is needed to confirm this benefit.

The clinical significance of reducing false-positive rates extends beyond mere diagnostic accuracy metrics. In emergency department settings, where radiologists must prioritize multiple urgent studies concurrently, high false-positive rates can lead to unnecessary patient recalls for repeat imaging or clinical correlation, thereby increasing both patient anxiety and healthcare costs. The 31% reduction in false positives directly translates to fewer false alarms requiring radiologist verification. By significantly reducing false-positive rates, our Modified Mask R-CNN has the potential to enhance workflow efficiency in high-volume clinical settings [22].

The potential for workflow integration in our model extends to several established clinical paradigms, including common implementation approaches such as triage prioritization, where AI automatically elevates PE-positive studies in radiologist worklists, concurrent reading, where AI results are displayed alongside images with heatmaps highlighting suspected emboli, and automated Pulmonary Embolism Response Team (PERT) activation for high-acuity cases [32]. The enhanced specificity for clinically significant emboli (volume ≥ 1000 mm^3^) ensures that when the AI flags a large embolus, radiologists can have greater confidence in its validity, facilitating rapid treatment decision-making.

It should be emphasized that while we employed a 1000 mm^3^ volume threshold for performance evaluation and comparison with prior studies, this should not be interpreted as a clinical recommendation that emboli below this threshold are clinically insignificant. In everyday clinical practice, management decisions regarding pulmonary embolism are rarely based solely on embolus volume [17]. Smaller emboli may still be clinically significant and warrant treatment depending on the patient’s overall clinical condition, underlying comorbidities, and cardiopulmonary reserve [17]. For instance, patients with limited cardiac function, chronic obstructive pulmonary disease, or prior venous thromboembolism may experience significant hemodynamic consequences from emboli that would be well tolerated in otherwise healthy patients. Therefore, clinical judgment integrating patient-specific factors should always supersede algorithmic volume thresholds in individual patient management decisions.

The clinical implications of the observed reduction in sensitivity also warrant careful consideration. The Modified Mask R-CNN demonstrated a modest decrease in patient-level sensitivity from 0.920 to 0.892 (internal) and from 0.96 to 0.88 (external), representing a trade-off for substantially improved specificity and PPV. Current clinical guidelines acknowledge the uncertainty regarding the optimal management of isolated subsegmental pulmonary embolism, with some advocating surveillance rather than anticoagulation in low-risk patients without proximal deep vein thrombosis [17]. If the sensitivity reduction predominantly affects small subsegmental emboli, as suggested by our volume-stratified analysis given the maintained high specificity (0.859) for emboli ≥1000 mm^3^—the clinical impact may be limited. However, missing lobar or central emboli, which are associated with hemodynamic compromise and adverse outcomes [17], is clinically concerning. Therefore, considering its high specificity, our model may be optimally positioned as a highly reliable ‘rule-in’ tool to prioritize urgent cases.

### 4.3. Limitations

This study has several limitations that merit consideration. First, the retrospective design and single-center training data may have introduced selection biases related to patient demographics, uniform scanner protocols, and institutional imaging practices [2,4]. Although external testing was conducted using a heterogeneous cohort (RSNA dataset), future multicenter studies with larger, multinational populations are necessary to ensure generalizability across broader clinical and technical environments.

Second, although the model demonstrated significant reductions in overall false positives, its performance in subsegmental regions, characterized by small vessel diameters (<2 mm) and contrast heterogeneity, remained suboptimal [10,11]. This limitation is consistent with the challenges reported in multi-detector CT-based PE detection studies, where partial volume effects and motion artifacts disproportionately affect small vessel analysis.

Third, we did not quantify reading time or workflow efficiency metrics, precluding an assessment of the AI system’s potential to accelerate triage in high-volume settings. Prior studies have demonstrated that AI-assisted workflows can reduce CTPA interpretation delays [22], even without improving senior radiologist accuracy, which is a critical consideration in the emergency department.

Fourth, we excluded CTPA examinations with suboptimal contrast enhancement (main pulmonary artery attenuation ≤ 200 HU) or significant motion artifacts. While this exclusion was necessary to ensure standardized training conditions and reliable ground-truth annotations, it represents an important limitation because these technically challenging examinations are precisely the cases that generate the greatest diagnostic uncertainty in daily clinical practice [16]. Such examinations are particularly common in emergency department settings, where patient cooperation may be limited, respiratory motion artifacts are frequent, and contrast bolus timing may be suboptimal due to hemodynamic instability [3]. Consequently, the current results may not be directly generalizable to these suboptimal studies, and a dedicated investigation of model performance in technically inadequate CTPA images remains an important area for future research.

Fifth, we did not perform a detailed characterization of false-negative cases stratified by anatomical site, although qualitative review suggested that missed emboli were predominantly small. While the observed reduction in patient-level sensitivity represents a trade-off for improved specificity, the clinical significance of this trade-off depends on the nature of the missed emboli. From a clinical perspective, missing isolated subsegmental PE may be more acceptable, given the ongoing debate regarding their treatment necessity and relatively low hemodynamic impact [17], whereas missing lobar or central emboli is more concerning because of their association with hemodynamic instability and adverse outcomes [17]. Future studies should include detailed false-negative analyses distinguishing between subsegmental, segmental, and lobar/central emboli to better characterize the clinical implications of this sensitivity-specificity trade-off and ensure that the FPR module does not disproportionately filter clinically significant proximal emboli.

### 4.4. Strengths of the Present Study

Despite these limitations, the present study has several notable strengths. Firstly, we developed and validated our model using real-world clinical data from consecutive patients, thereby ensuring exposure to a comprehensive spectrum of clinical presentations and technical challenges encountered in routine practice, rather than curated datasets.

Secondly, we evaluated the model’s performance on both internal and external datasets, with the external cohort (RSNA PE challenge dataset) encompassing significant heterogeneity in scanner vendors and acquisition parameters, thus assessing the model’s robustness in realistic deployment conditions. The consistency of performance metrics across both cohorts demonstrates genuine generalizability rather than overfitting to institution-specific patterns.

Thirdly, the modular design facilitates future enhancements and adaptations to institutional preferences [32] without necessitating complete retraining of the model. This architectural flexibility allows individual healthcare institutions to adjust the decision threshold of the FPR module based on their specific clinical priorities, whether prioritizing sensitivity for high-acuity emergency triage or emphasizing specificity to minimize unnecessary anticoagulation in outpatient settings, a capability that single-stage end-to-end models cannot accommodate.

Finally, our volume-stratified analysis provides clinically relevant performance metrics focused on hemodynamically significant emboli, aligning model evaluation with clinical decision-making priorities. The enhanced specificity for large emboli ensures greater diagnostic confidence when the AI flags high-risk cases requiring urgent intervention, such as Pulmonary Embolism Response Team activation or consideration for advanced therapies beyond standard anticoagulation. Importantly, our significant improvement from an already reasonable baseline demonstrates practical value; the FPR module adds value even when the baseline detection is already reasonable, representing a more realistic scenario for institutional deployment than studies reporting improvements from poorly performing initial systems.

## 5. Conclusions

In conclusion, the findings suggest that the Modified Mask R-CNN framework yields improvements in diagnostic accuracy and specificity over the RPN-only model by achieving a substantial reduction in false-positive rates while maintaining comparable sensitivity. By incorporating a specialized false-positive reduction mechanism, this two-stage architecture may enhance the reliability and interpretability of automated pulmonary embolism detection, suggesting its potential utility as a clinical decision-support tool.

## Figures and Tables

**Table 1:** Patient baseline demographic and imaging characteristics.

Characteristics		All Patients (n = 303)	PE Positive (n = 163)	PE Negative (n = 140)	*p*-Value
**Demographics**					
Age (years)		63.2 ± 16.8	63.5 ± 16.4	62.9 ± 17.3	0.78
Gender					0.158
	Female	161	80 (49.7%)	81 (50.3%)	
	Male	142	83 (58.5%)	59 (41.5%)	
**Clinical Characteristics** **& Risk Factors, n (%)**					
Hypertension		150/303 (49.5%)	79/163 (48.5%)	71/140 (50.7%)	0.71
Diabetes mellitus		68/303 (22.4%)	34/163 (20.9%)	34/140 (24.3%)	0.48
Active Malignancy		41/303 (13.5%)	26/163 (16.0%)	15/140 (10.7%)	0.18
Recent Surgery		27/303 (8.9%)	19/163 (11.7%)	8/140 (5.7%)	0.07
**Laboratory**					
	D-dimer, mg/L (median [IQR])	0.75 [0.35–1.90]	1.20 [0.55–3.40]	0.42 [0.26–0.82]	<0.001
Scanner vendor, n (%)	**Imaging acquisition**				
					0.069
	Siemens Healthineers	57/303 (18.8%)	24/163 (14.7%)	33/140 (23.6%)	
	Philips	246/303 (81.2%)	139/163 (85.3%)	107/140 (76.4%)	

**Table 2:** Comparative diagnostic performance on the internal validation dataset (n = 303).

Metric	RPN-Only Model (95% CI)	Modified Mask R-CNN (95% CI)	Difference	*p*-Value
Patient-level sensitivity	0.920 (0.851–0.966)	0.892 (0.821–0.950)	−0.028	0.500 †
Patient-level PPV	0.650 (0.558–0.727)	0.718 (0.622–0.792)	0.068	<0.001
Lesion-level PPV	0.573	0.658	0.085	N/A
False positives per scan	0.331 (0.243–0.419)	0.228 (0.156–0.285)	−0.103	<0.001 ‡
Lesion-level sensitivity	0.884	0.870	−0.014	N/A
Patient-level specificity	0.800 (0.737–0.846)	0.859 (0.801–0.897)	0.059	<0.001 †

Values in parentheses are 95% confidence intervals. † Calculated using McNemar’s test. ‡ Calculated using Wilcoxon signed-rank test. PPV, positive predictive value.

**Table 3:** External validation on the RSNA cohort (n = 100; 50 PE+, 50 PE−): paired comparison between RPN-only Model and Modified Mask R-CNN.

Metric	RPN-Only Model	Modified Mask R-CNN	Difference	*p*-Value
Patient-level sensitivity	0.96 (0.842–0.987)	0.88 (0.750–0.948)	−0.08	0.125 †
PPV	0.52 (0.396–0.634)	0.76 (0.619–0.854)	0.24	<0.001
False positives per scan	1.1 (0.928–1.272)	0.34 (0.260–0.420)	−0.76	<0.001 ‡
Lesion-level sensitivity	0.97	0.92	−0.05	N/A
Patient-level specificity	0.59 (0.458–0.704)	0.84 (0.731–0.916)	0.25	<0.001 †

Values in parentheses are 95% confidence intervals. † Calculated using McNemar’s test. ‡ Calculated using Wilcoxon signed-rank test. PPV, positive predictive value.

**Table 4:** Performance of the Modified Mask R-CNN Model Across Different Volume Thresholds for False-Positive Reduction.

Volume Threshold	Patient Sensitivity	Patient Specificity	PPV	False Positives per Scan
300	0.86	0.7	0.58	0.565
500	0.88	0.82	0.65	0.355
750	0.91	0.88	0.7	0.231
1000	0.88	0.88	0.72	0.168
1500	0.91	0.93	0.76	0.103
2000	0.93	0.94	0.76	0.089

Volume thresholds in mm^3^. All metrics calculated for the Modified Mask R-CNN model on internal validation dataset.

## Data Availability

The internal clinical data presented in this study are not publicly available because of patient privacy restrictions. The external test dataset is publicly available at the RSNA PE Challenge website (https://www.rsna.org/education/ai-resources-and-training/ai-image-challenge/rsna-pe-detection-challenge-2020). (accessed on 15 October 2025).
